# Commentary: The Social Dilemma of Autonomous Vehicles

**DOI:** 10.3389/fpsyg.2017.00808

**Published:** 2017-05-24

**Authors:** Rose Martin, Ivaylo Kusev, Alex J. Cooke, Victoria Baranova, Paul Van Schaik, Petko Kusev

**Affiliations:** ^1^Department of Psychology, Kingston University LondonLondon, UK; ^2^Department of Management, Huddersfield Business School, University of HuddersfieldHuddersfield, UK; ^3^Department of Economic Sociology, University of National and World EconomySofia, Bulgaria; ^4^Department of Psychology, Lomonosov Moscow State UniversityMoscow, Russia; ^5^Department of Psychology, Sport and Exercise, Teesside UniversityMiddlesbrough, UK

**Keywords:** autonomous vehicles, utility theory, moral dilemmas, uncertainty, accessibility

An autonomous vehicle (AV) car with 1 passenger (e.g., the car owner) inside is traveling within the speed limit down the road. However, unexpectedly, 10 pedestrians have appeared in its path and it is too late for the car to brake. The car must either save the passenger by driving into the 10 pedestrians and killing them, or save the 10 pedestrians by swerving into a barrier and killing the passenger. Should the AV algorithm be programmed to save the passenger, or to save the greater number of people? This question is of great importance to the AV industry, policy makers, the potential buyers, and the general public.

Recent research (Bonnefon et al., 2016) has investigated how humans judge the morality of the two AV algorithms—a utilitarian (saving the greater number of lives; Bentham, 1970) and a non-utilitarian passenger-protective (saving the passenger). Using moral dilemma scenarios in which an AV is programed to be utilitarian or passenger-protective, participants were required to rate (on a 0–100 slider) “what action they thought was the most moral,” Bonnefon et al. (2016) found that participants rated the utilitarian algorithm (e.g., sacrificing 1 passenger to save 10 pedestrians) as the moral course of action. However, when the respondents were asked to rate on a scale the likelihood of buying a car with each of the algorithms (to what extent they are inclined), they indicated higher likelihood of purchasing the passenger-protective algorithm than the utilitarian one. This surprising result demonstrates what appears to be a social dilemma—an agent temptation to act in accordance with self-interest (Bonnefon et al., 2016), which often results in the worst outcome for all individuals involved, including the decision-maker (Dawes, 1980; Kollock, 1998). The authors have not explained theoretically the results from this social dilemma; yet they discounted the possibility of uncertainty (e.g., the possibility that people may not be aware or have access to the utilitarian actions and their consequences; Kusev et al., 2016; Zhao et al., 2016). Therefore, the aim of this paper is to provide an insight (theoretical and methodological) and explanation for the surprising reversals of the moral utilitarian preferences reported in Bonnefon et al. (2016).

Here, we argue that methodological issues in Bonnefon's et al. (2016) research may have induced uncertainty amongst participants. Accordingly, it is plausible that the difference in response to the two questions (what action they thought was the most moral, and to what extent are they are inclined to purchase an AV with each algorithm) is caused by the restricted accessibility of moral utilitarian information. For example, respondents may not realize that a car purchaser is also inevitably a pedestrian too. We suggest that full utilitarian descriptions provide accessibility to utilitarian tasks and their consequences and can eliminate the conflicting responses to these two questions.

Psychological uncertainty has been found to account for respondents' differences in utilitarian choice for morally sensitive scenarios (Kusev et al., 2016); comprehensive moral tasks and questions reduced decision uncertainty and boosted utility maximization. Kusev et al. (2016) argued that in moral decision-making tasks (the trolley and footbridge dilemma; Thomson, 1985; Greene et al., 2001) participants are given (i) a partial moral task description which outlines what will happen should they throw the switch/push the stranger, and (ii) asked a partial appropriateness of action question for only one of the two possible moral actions (yes/no answers). Hence, the respondents are left to infer what will happen should they refrain from this action and asked to judge the appropriateness of only one of the actions. Thus, the moral dilemma and question contain only “partial utilitarian descriptions,” inducing uncertainty.

Accordingly, we argue that all of the scenarios presented by Bonnefon et al. (2016) contained partial utilitarian information, inducing decision uncertainty amongst respondents. For instance, in some of the scenarios each respondent is required to imagine themselves as a passenger inside the car and are therefore presented with only one side of the situation. We propose that if the respondents imagine themselves as pedestrians as well, uncertainty may be reduced as respondents would be able to access both possibilities—being a passenger and a pedestrian. For instance, being in the car and benefiting from the passenger-protective algorithm does not expose the respondents to the greater danger/risk of other cars employing the same algorithm when they are not in the car (e.g., as pedestrians).

In addition to the partial information in the scenarios presented by Bonnefon et al. (2016), we further argue that the questions the authors claim to produce conflicting results in studies 3, 4, and 6 do not fully account for the willingness to buy an AV car. In the experiments participants were presented with a moral scenario where an AV can either be programmed to be utilitarian, passenger-protective, or select either option at random. This scenario was followed by questions, one of which required the participants to rate their relative willingness to buy an AV for themselves—“How would you rate your relative willingness of having an AV with each of these algorithms?.” The results revealed that participants preferred to purchase the passenger-protective AV, which once again conflicted with their general preference for utilitarian AV. Due to this conflict, our ongoing research aims to comprehensively understand utilitarian behavior by providing respondents with two moral questions regarding their willingness to purchase an AV, and their willingness for other people to purchase an AV:

“Please rate how willing you would be to purchase an AV that is programmed with each of these algorithms”

and

“Please rate how willing you would be for other people to purchase an AV that is programmed with each of these algorithms.”

It is plausible that full accessibility to moral tasks and questions reduces decision uncertainty and maximizes utility in moral decision-making with AVs. In our proposal, we argue that utility maximization can be increased by enabling participants to imagine themselves as not only as a passenger of an AV, but also as a pedestrian, and measure their judgments appropriately.

## Author contributions

RM drafted and revised the manuscript. PK (corresponding author) initiated and revised the general commentary. IK, AC, VB, and PV provided feedback and suggestions. All authors approved the final version of the manuscript for submission.

### Conflict of interest statement

The authors declare that the research was conducted in the absence of any commercial or financial relationships that could be construed as a potential conflict of interest.
